# Diagnostic Value of Ultraviolet-Induced Fluorescence Dermoscopy in Cutaneous Fungal Infection

**DOI:** 10.7759/cureus.110437

**Published:** 2026-06-08

**Authors:** Marwa Elbadawy, Raiyaan Sakib

**Affiliations:** 1 Dermatology, Fakeeh University Hospital, Dubai, ARE; 2 Family Medicine, Fakeeh University Hospital, Dubai, ARE

**Keywords:** dermoscopy, diagnostic efficacy, fungal, koh, uvf

## Abstract

Introduction

Fungal skin infections are extremely common worldwide. The diagnosis of cutaneous fungal infections depends on clinical examination and laboratory confirmation using potassium hydroxide (KOH) preparation. Ultraviolet-induced fluorescent dermoscopy (UVF) may provide additional diagnostic clues for dermatologists. UVF dermoscopic findings were compared with the reference standard KOH test results for suspected cutaneous fungal infections.

Objectives

The aim of the present study was to examine UVF dermoscopy reliability in aiding the diagnosis of cutaneous fungal infections.

Methods

This is a retrospective observational study based on data review, laboratory results, and image analysis. Patients with suspected fungal infections were subjected to UVF examination and KOH tests. For each eligible patient, demographic and clinical data were extracted, including age, sex, and a unique patient ID or record number.

Results

The present study included 150 patients with various types of cutaneous fungal infections, who were examined by the UVF dermoscopy and KOH wet mount. The sensitivity was 98.63%, while the specificity was 80.52% in comparison with the KOH examination.

Conclusions

Ultraviolet fluorescent dermoscopy demonstrated high sensitivity and high negative predictive value, supporting its role as a rapid adjunctive screening tool for superficial fungal infections. UVF-guided sampling may facilitate more accurate KOH examination. However, UVF dermoscopy should be considered complementary to conventional mycological investigations rather than a replacement.

## Introduction

Cutaneous fungal infections caused by dermatophyte and non-dermatophyte fungi represent significant challenges to global public health [1]. The diagnosis of superficial fungal infections depends on clinical examination followed by laboratory confirmation. The potassium hydroxide (KOH) preparation is a simple bedside test that involves collecting a sample by scraping keratin from an active border area of the rash or nail to be treated with KOH and examined microscopically for fungal elements. KOH microscopy is a widely used diagnostic method with good sensitivity and specificity. Despite these methods, there is a need for more immediate tools to guide treatment decisions [2].

The use of dermoscopy for non-neoplastic dermatoses has expanded, with improvement in diagnostic accuracy [3]. Ultraviolet-induced fluorescent dermoscopy (UVF) may provide the dermatologist with additional diagnostic clues [4]. UVF dermoscopy is a new technique that utilizes a UV light source (365 nm) perceived by the observer through fluorochromes that emit UV-excited luminescence in a phenomenon known as the Stokes shift [5].

UVF dermoscopic findings will be compared with the reference standard KOH test results for suspected cutaneous fungal infections. The aim of the present study is to provide evidence on UVF dermoscopy reliability in aiding the diagnosis of cutaneous fungal infections as an additional rapid and non-invasive diagnostic tool in patient management.

## Materials and methods

Study design and setting

This retrospective observational study was based on data review, laboratory KOH results, and image analysis. The study was conducted at the Dermasurge Department of Fakeeh University Hospital, Silicon Oasis, Dubai, UAE. Data was collected from the 5th of October 2023 until the 30th of May 2025.

Participants

Patients with suspected fungal infections based on UVF examination and KOH tests were included in the study. The inclusion criteria were that the diagnosis was made by KOH test result and high-quality dermoscopic imaging captured at 10X with both polarised and UV light (dry contact). Patients who received treatment within 6 weeks before examination were excluded. Patients with unsatisfactory imaging quality or inconclusive KOH test results were excluded. For each eligible patient, demographic and clinical data were extracted, including age, sex, and unique patient ID or record number (which was anonymized).

Imaging and evaluation

**Figure 1:**
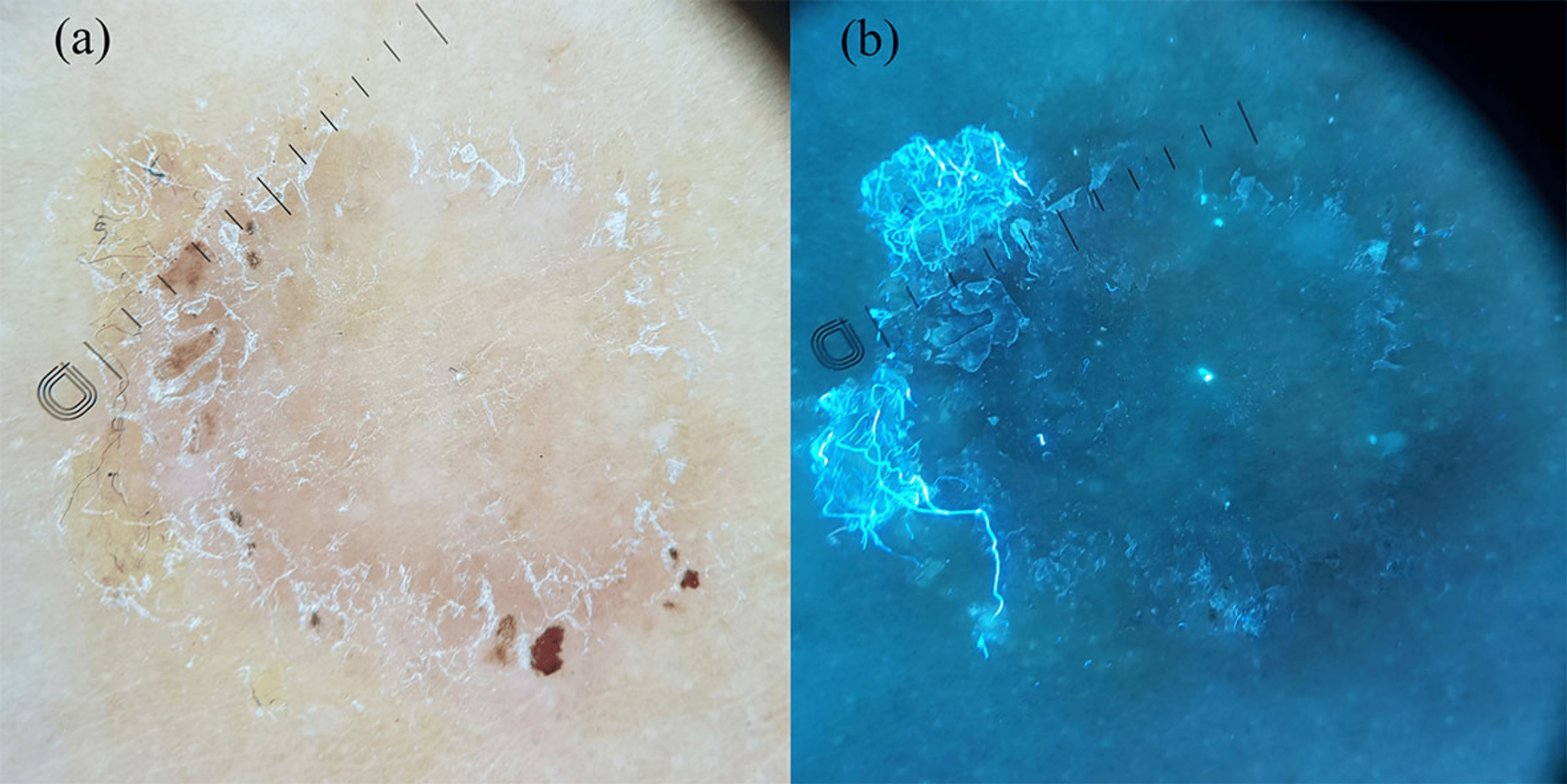
Tinea Corporis (a) Polarized dermoscopic image showing a circinate lesion on the face of a 2-year old patient (b) UVF dermoscopic image revealing green blue fluorescence at the active border. UVF: ultraviolet-induced fluorescent

**Figure 2:**
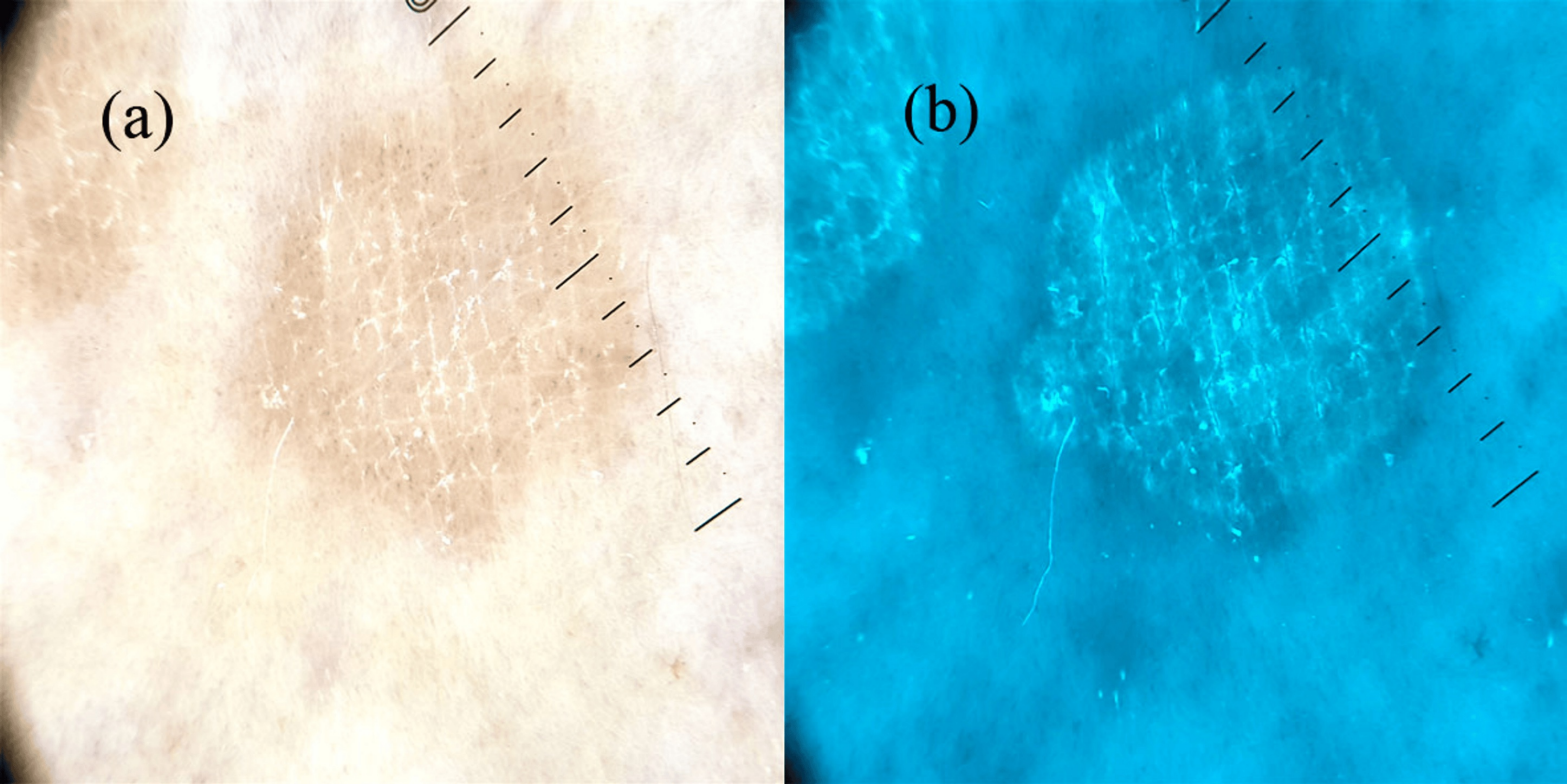
Pityriasis Versicolor (a) Polarized dermoscopic image showing a light brown lesion (b) UVF dermoscopic image showing faint yellow fluorescence throughout the whole lesion. UVF: ultraviolet-induced fluorescent

**Figure 3:**
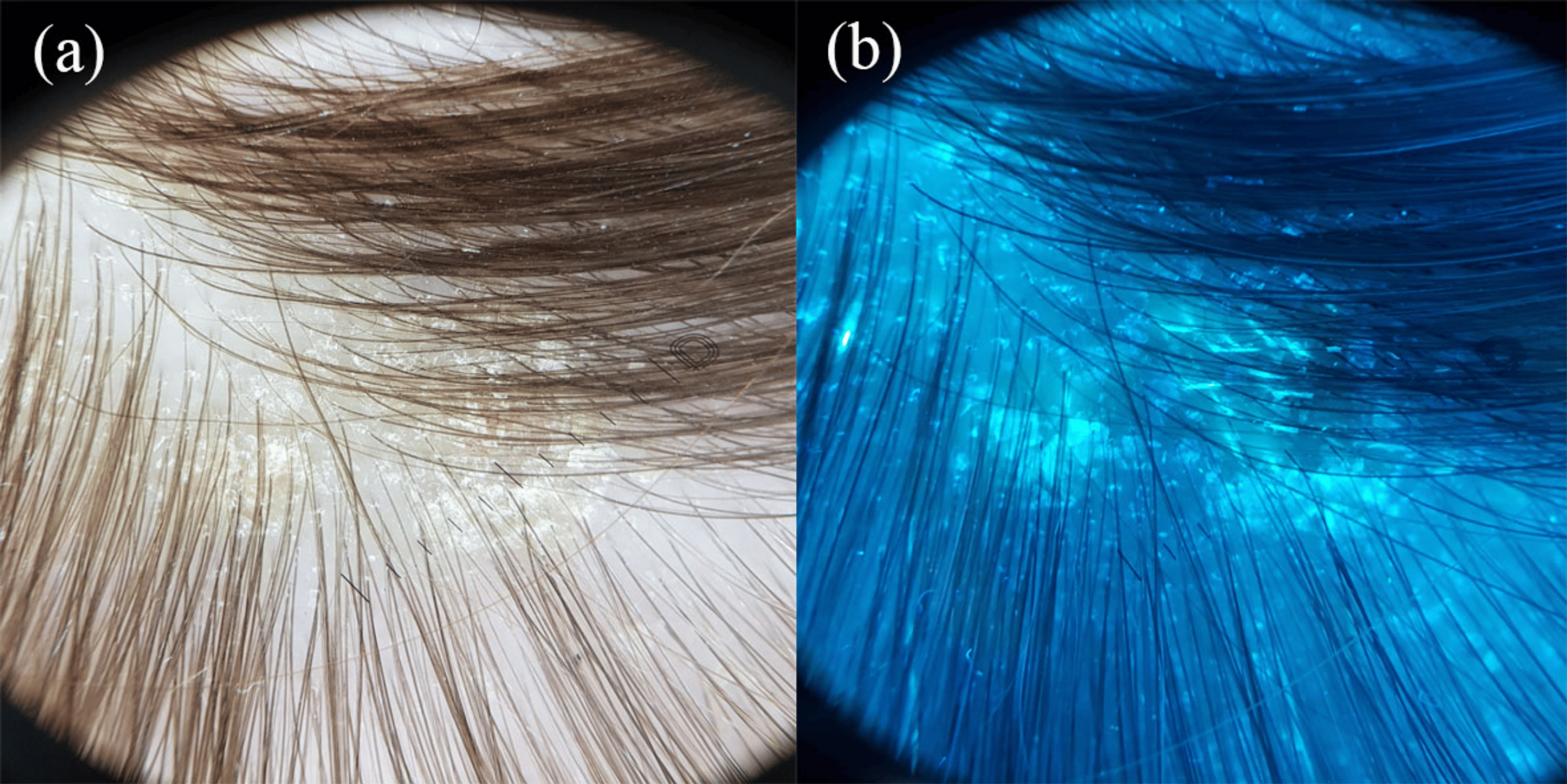
Tinea Capitis (a) Polarized dermoscopic image showing perifollicular scales (b) UVF dermoscopic image showing bright-green fluorescence at the affected scalp and on the infected hair shafts. UVF: ultraviolet-induced fluorescent

**Figure 4:**
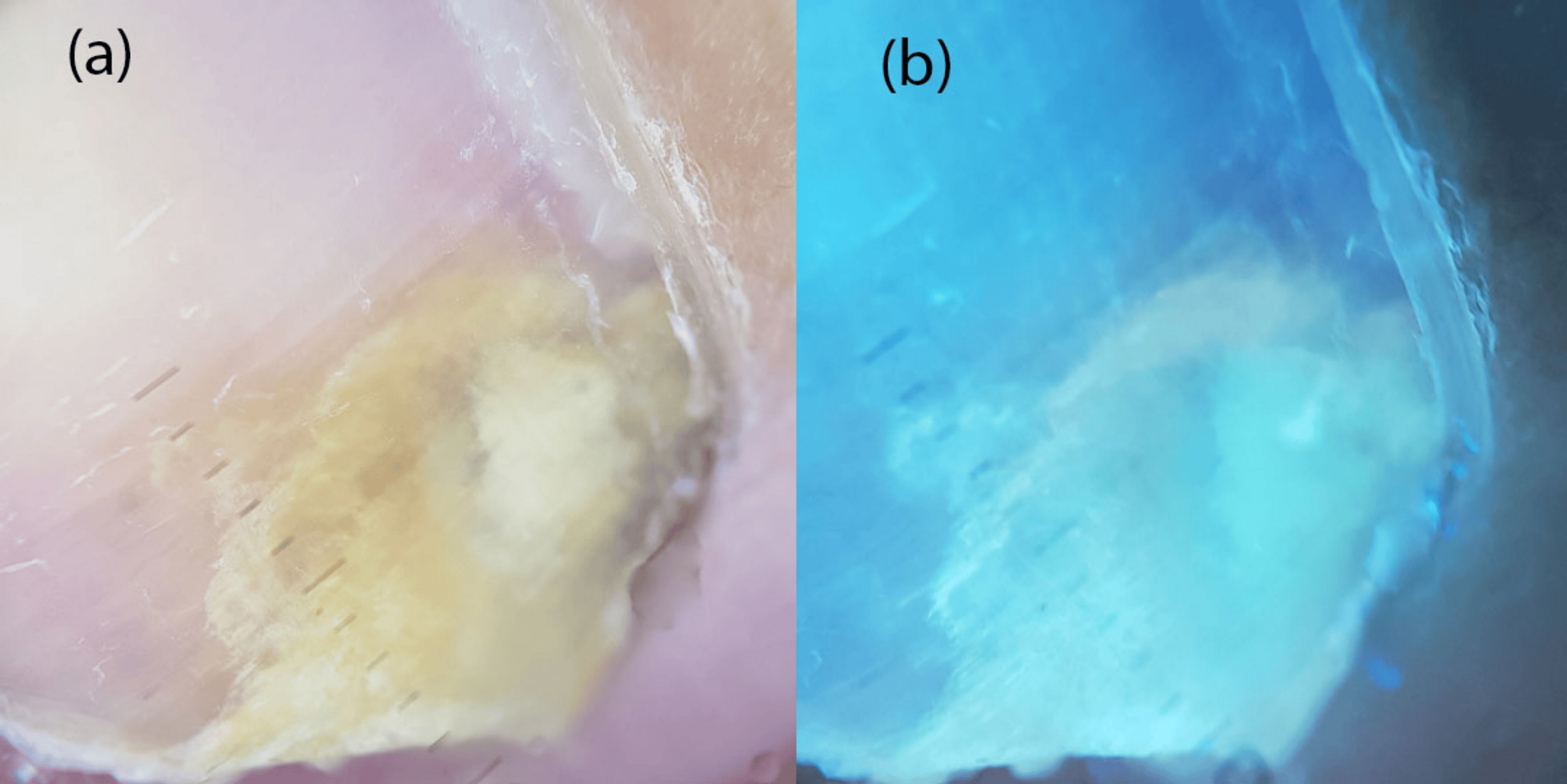
Tinea Unguium (a) Polarized dermoscopic image showing yellowish discoloration of the nail plate with roughness and thickening (b) UVF dermoscopic image showing pale distal fluorescence at the affected nail. UVF: ultraviolet-induced fluorescent

All patients were examined using a handheld dermatoscope, Dermlite DL5 (San Juan Capistrano, CA, United States), coupled with a smartphone. Two independent investigators evaluated all images and were blinded to the KOH results. UVF patterns were interpreted based on previously published dermoscopic and Wood’s lamp-correlated fluorescence characteristics reported in the literature. Previously described fluorescence patterns included blue fluorescence in Malassezia folliculitis and red follicular fluorescence in pityriasis versicolor [6]. Other types of fluorescence, such as bright-white fluorescence (accumulated scales), bright-green fluorescence (hair shafts), and bright-white fluorescence disappearance at the white bands of the barcode-like hairs, have also been observed in tinea capitis (Figures 1-4) [7].

Direct microscopy

Microscopic examination of the scales obtained by skin scraping was performed after treatment with 10% KOH for the detection of fungal elements. Scales were collected by scraping the lesions with sterile number 15 scalpel blades at the borders of the lesions. Two drops of 10% KOH were added to glass slides with calcofluor dye for fungal elements. A coverslip was placed over it, and the preparation was examined under the 400X objective of the ZEISS fluorescent microscope [8] (Figures 1-4). KOH results are typically reported as positive (fungal hyphae/spores) or negative (no fungal elements). The study was approved by the Institutional Review Board of the Fakeeh University Hospital, Dubai, UAE (approval number FUH-RES-034-025).

Statistical analysis

The clinical, polarized dermoscopy, and UVF dermoscopy findings were compared with the results of the KOH examination and classified as either true positive, true negative, false positive, or false negative.

Sensitivity, specificity, positive predictive value (PPV), negative predictive value (NPV), and overall diagnostic accuracy of UVF dermoscopy were calculated using KOH microscopy as the reference standard. Receiver Operating Characteristic (ROC) curve was generated and area under the curve (AUC) was calculated with exact 95% confidence intervals [9]. All analyses were performed using SPSS software (version 22, IBM, Armonk, NY). Cohen’s kappa was used as a measure of agreement rather than diagnostic accuracy, complementing sensitivity and specificity analyses.

## Results

**Figure 5:**
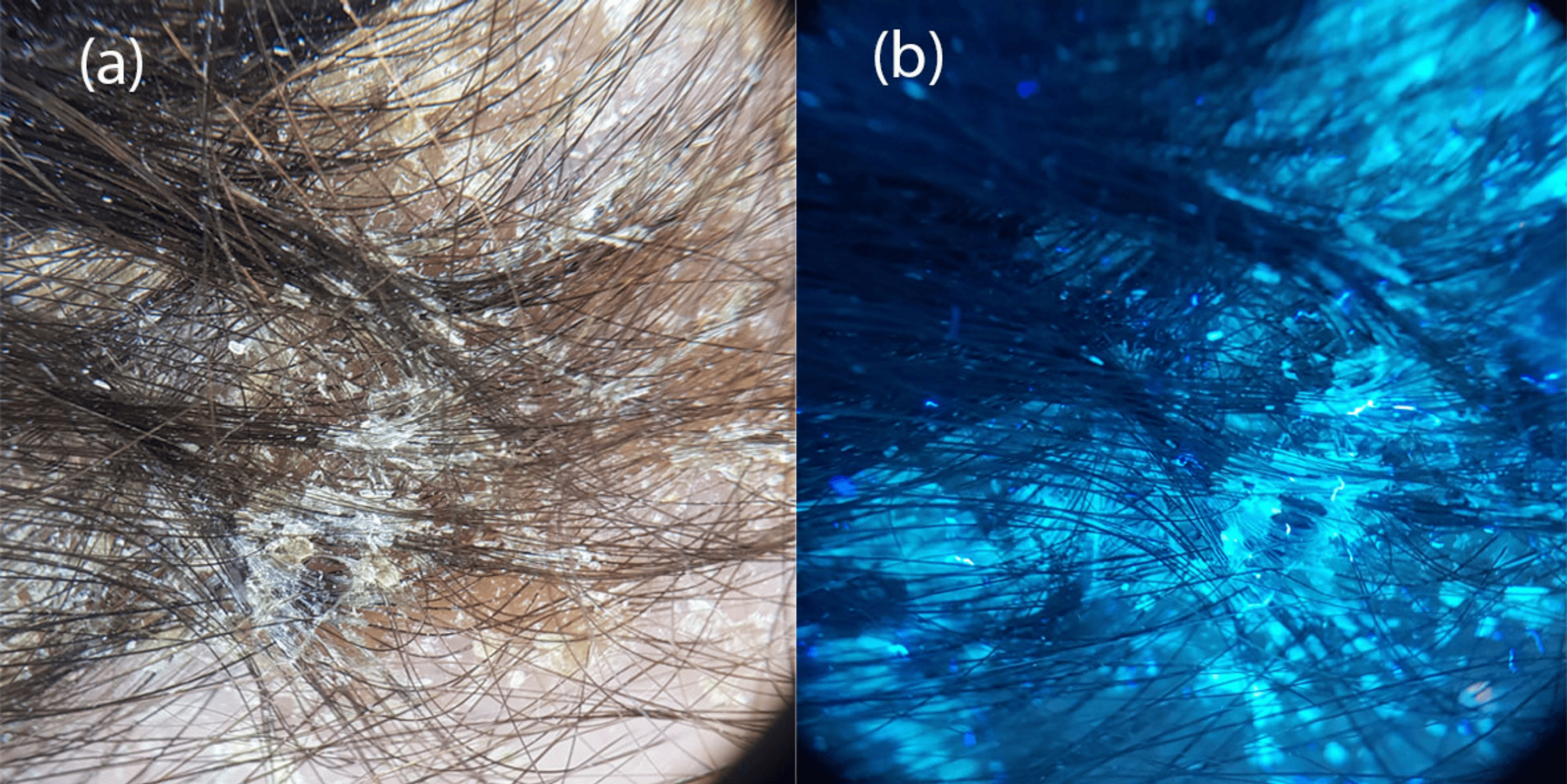
False positive staphylococcal infection of the scalp (a) Polarized dermoscopic image showing a yellowish crust and mild oozing with no hair shaft involvement, (b) UVF dermoscopic image showing bright yellow fluorescence at the affected scalp. UVF: ultraviolet-induced fluorescent

**Figure 6:**
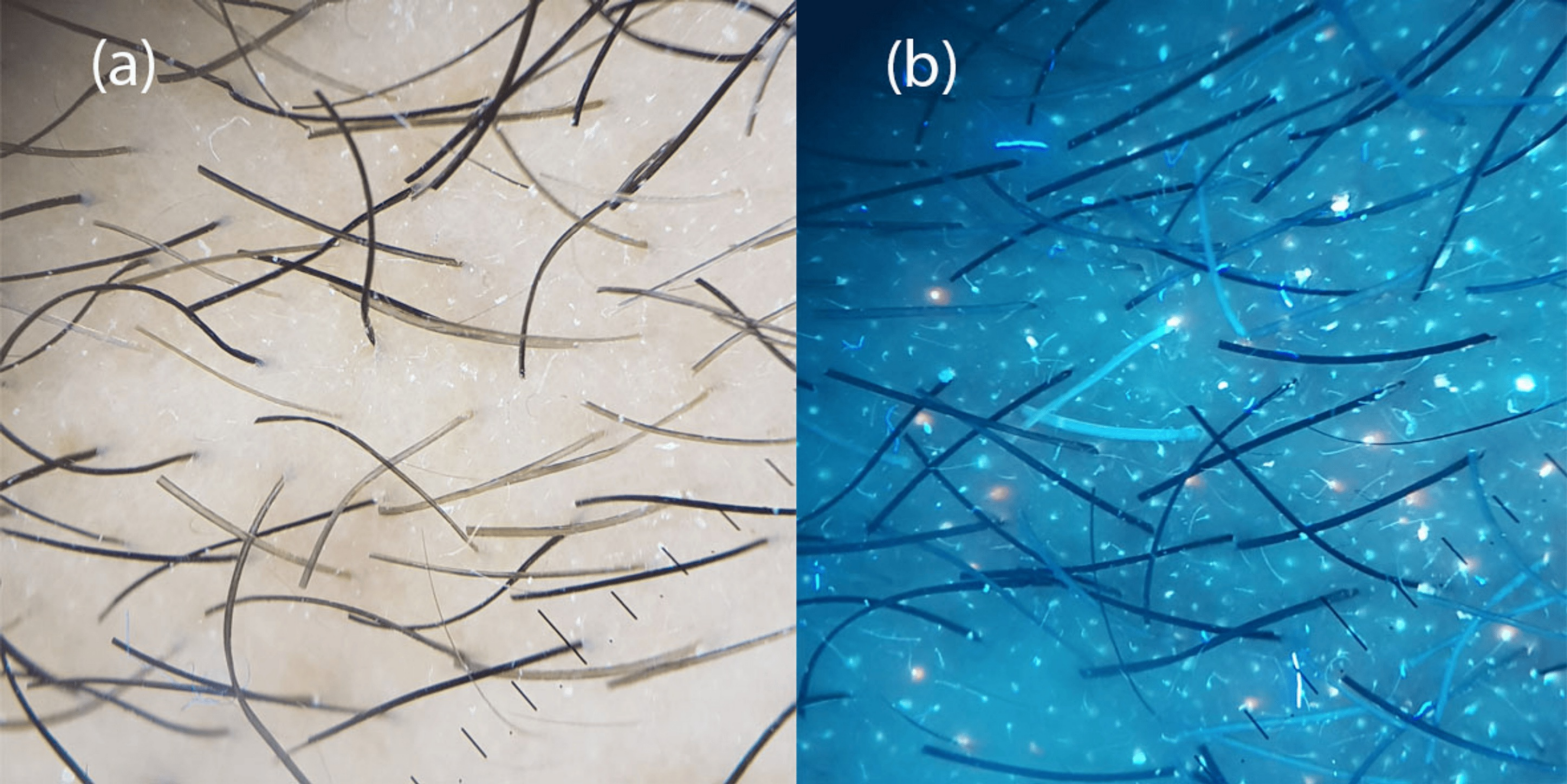
False positive pink follicular fluorescence of the beard area (a) Polarized dermoscopic image showing minimal scales, (b) UVF dermoscopic image showing bright pink follicular fluorescence. UVF: ultraviolet-induced fluorescent

**Figure 7:**
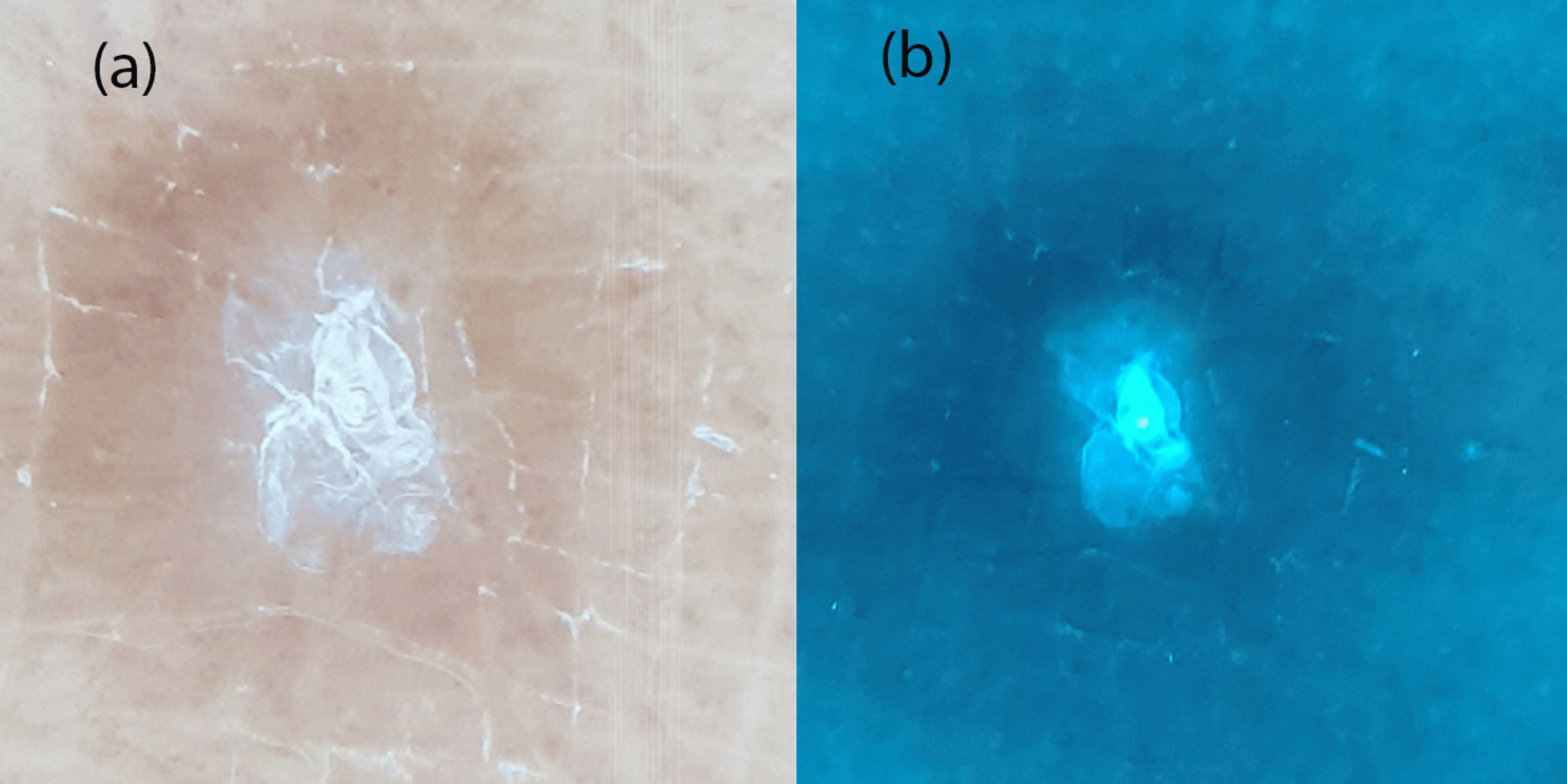
False positive bright fluorescence on the leg in guttate psoriasis lesion (a) Polarized dermoscopic image of silvery white scales on an erythematous background, (b) UVF dermoscopic image showing bright green fluorescence. UVF: ultraviolet-induced fluorescent

The present study included 150 patients (63 males and 87 females), with a mean age of 26.72 years with various types of true positive cutaneous fungal infections, including tinea corporis (30) (Figure 1), pityriasis versicolor (20) (Figure 2), tinea cruris (7), tinea capitis (6) (Figure 3), tinea unguium (5) (Figure 4), tinea pedis (4), and one patient with candidiasis with no fluorescence (Table 1). False positive results due to staphylococcal infection fluorescence (Figure 5), follicular fluorescence of *Cutibacterium *species (Figure 6), and guttate psoriasis (Figure 7).

**Table 1:** Types, percentages, and fluorescence of fungal infections UVF: ultraviolet-induced fluorescent

Type	Number	Percent	UVF dermoscopy
Tinea corporis	30	20.00%	Green fluorescent scales
Pityriasis versicolor	20	13.33%	Pale yellow fluorescence
Tinea cruris	7	4.67%	Bright yellow fluorescence
Tinea capitis	6	4.00%	Bright green fluorescence and hair
Tinea unguium	5	3.33%	Yellow fluorescence of the nail plate
Tinea pedis	4	2.67%	Yellow-green fluorescence of scales
Candidiasis	1	0.67%	No fluorescence, KOH positive

Agreement between UVF dermoscopy and KOH microscopy was assessed using Cohen’s kappa coefficient. The overall agreement was substantial (κ = 0.79), indicating good concordance between the two diagnostic modalities.

This study compared two different methods for diagnosing superficial cutaneous fungal infections. As the KOH examination was used as the reference standard in the absence of fungal culture or molecular confirmation, UVF dermoscopy was compared to it. Of the 150 patients studied, UVF was positive in 87 (58%) patients, while KOH examination was positive in 73 (48.67%) patients. Negative results were observed in 63 (42 %) patients with UVF dermoscopy and 77 (51.33%) patients with KOH examination (Table 2).

**Table 2:** Diagnostic performance of ultraviolet-induced fluorescence (UVF) dermoscopy compared with potassium hydroxide (KOH) microscopy in cutaneous infections

		KOH scraping result		Total
		Positive	Negative	
UVF	Positive	72	15	87
	Negative	1	62	63
Total		73	77	150

**Figure 8:**
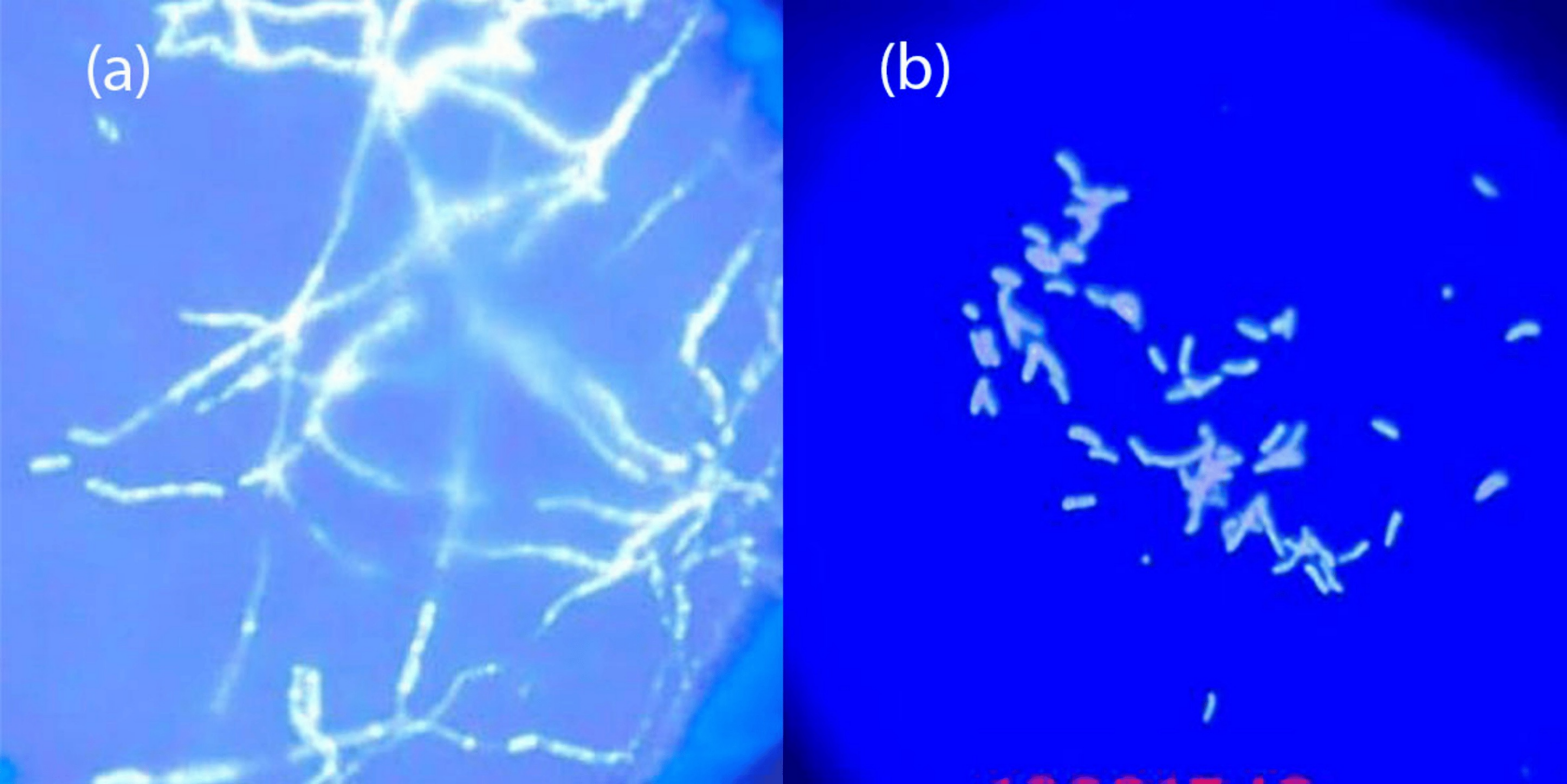
Direct microscopy using KOH preparation (a) Long hyphae of fungal infection, (b) yeast cells and pseudo hyphae (“spaghetti and meatball” appearance) of *Malassezia spp.* ZEISS fluorescent microscope (400x magnification; Carl Zeiss Microscopy GmbH, Jena, Germany). KOH: potassium hydroxide

**Figure 9:**
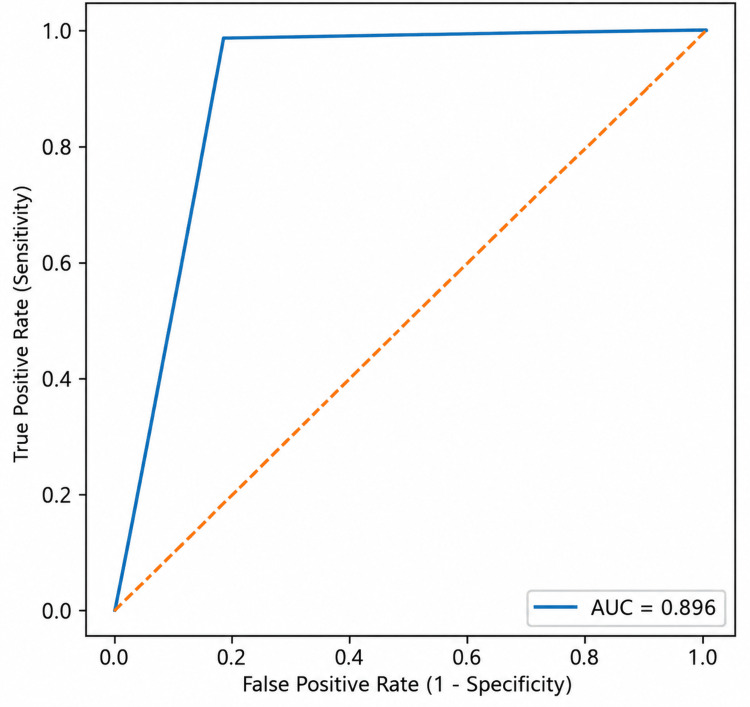
Receiver operating characteristic (ROC) curve of ultraviolet-induced fluorescence (UVF) dermoscopy compared with potassium hydroxide (KOH) microscopy in the diagnosis of superficial fungal infections. The area under the curve (AUC) was 0.896, indicating very good diagnostic performance.

Diagnostic efficacy of UVF dermoscopy compared with KOH examination in superficial cutaneous fungal infections is summarized in Table 3. The sensitivity of UVF dermoscopy was 98.63% (95% CI, 92.60%-99.97%), whereas specificity was 80.52% (95% CI, 69.91%-88.67%) compared with KOH examination (Figure 8). The positive predictive value (PPV) was 82.76% (95% CI, 73.16%-90.02%), while the negative predictive value (NPV) was 98.41% (95% CI, 91.47%-99.96%). Overall diagnostic accuracy was 89.3%. The area under the receiver operating characteristic curve was 0.896 (Figure 9).

**Table 3:** Performance characteristics of UVF dermoscopy in the evaluation of superficial fungal infections UVF: ultraviolet-induced fluorescent

Diagnostic parameter	Value (%)	95% Confidence Interval
Sensitivity	98.63	92.60–99.97
Specificity	80.52	69.91–88.67
Positive Predictive Value (PPV)	82.76	73.16–90.02
Negative Predictive Value (NPV)	98.41	91.47–99.96
Overall Diagnostic Accuracy	89.30	—

## Discussion

Cutaneous fungal infections usually show characteristic fluorescence at specific wavelengths due to changes in skin pigment caused by colonization of the stratum corneum by a lipophilic fungus [6,10]. The Wood’s lamp is a conventional, simple method for identifying such infections [6]. However, UVF dermoscopy is a novel, non-invasive, and convenient technique that achieves better visualization of such fluorescence. Tang et al. [11] first reported the UVF dermoscopy findings in fungal infection secondary to chemical pteridine produced by some Microsporum species. Infections should be confirmed by direct microscopy using 10% potassium hydroxide to demonstrate hyphae, pseudohyphae, and blastoconidia [12].

The present study demonstrates that UVF dermoscopy may show clear findings that aid in facilitating the differential diagnosis of clinically similar fungal and yeast infections based on the presence or absence of specific skin chromophores produced by UV light. In pityriasis versicolor, the fungus produces pityrialactone, a fluorescent bisindole compound that produces yellow-orange to green-yellow fluorescence on exposure to Wood’s light [13]. In tinea capitis, a high positive green fluorescence rate could be detected in the case of Microsporum canis infection [14], while in tinea capitis caused by Trichophyton spp, no fluorescence was detected in a previous report [15]. These findings could be useful not only in the diagnosis of fungal infections but also in aiding the recognition of the possible species of the fungus using the trichoscopic criteria of tinea capitis, such as comma hairs, corkscrew hairs, zigzag hairs, Morse code-like hairs, broken hairs, and black dots in the absence of fluorescence [16].

This study compared UVF dermoscopy with KOH examination in patients with various types of fungal infections. In the present study, the sensitivity of UVF dermoscopy compared to KOH examination was 98.63%, and the specificity was 80.52%. Therefore, UVF dermoscopy may serve as a screening tool for fungal infections. To date, few studies are available regarding the diagnostic efficacy of UVF dermoscopy compared to KOH. However, if we compare UVF dermoscopy with Wood’s lamp for fungal infections, UVF dermoscopy shows higher sensitivity and lower specificity. This could be attributed to dermoscopy fluorescence detected in cases of bacterial infections, eczema, psoriasis, and other scaly conditions. The false-positive results can be attributed to co-existing bacterial infections in some cases, with Staphylococcus aureus, which may emit yellow fluorescence in addition to polarized dermoscopic findings of follicular-based pustules, a finding noticed in one of our patients and confirmed with bacterial culture. This finding was also observed by Couppoussamy and Devanda [17], who reported follicular and perifollicular findings in chronic superficial folliculitis.

The pink fluorescence of foot lesions can be attributed to Corynebacterium foot intertrigo, which has been observed in a previous study [4]. Facial false-positive fluorescence can be attributed to coproporphyrin III/ protoporphyrin IX production by Cutibacterium acnes, which may explain false-positive results that may be detected by Wood’s lamp [18]. Furthermore, red fluorescence has been previously observed using UVF dermoscopy in psoriasis and was positively correlated with psoriasis severity [19]. In our study, a case of guttate psoriasis showed bright greenish fluorescence, which was false-positive fluorescence, and KOH revealed negative fungal infection. The false-negative UVF was detected only in 1 case of candidiasis, which is in accordance with Errichitti et al [4], who noticed the absence of fluorescence in candidiasis as well.

Regarding the accuracy of UVF dermoscopy, the ROC analysis demonstrated very good diagnostic performance with an AUC of 0.896. This finding is the result of the association of fluorescent findings with specific chromophores produced in most cutaneous fungal infections, in addition to the value of polarized light-based examination without the requirement of a dark room to yield fluorescence on examination.

Ultraviolet-induced fluorescent dermoscopy is not only a useful adjunctive screening tool for cutaneous fungal infections for cutaneous fungal infections, but also helpful in detecting subclinical infection with a high sensitivity rate. Furthermore, UVF dermoscopy-guided sampling for KOH examination aids in highlighting a more accurate site to collect specimens to yield positive results. The high sensitivity (98.41%) of UVF dermoscopy renders it suitable as a screening test for suspected cutaneous fungal infections.

This study has several limitations. First, the retrospective design may have introduced selection bias and variability in image quality and clinical documentation. Second, although expert dermatologist interpretation and KOH microscopy were used as reference standards, fungal culture or polymerase chain reaction (PCR) confirmation was not available. Third, variability in smartphone camera quality, lighting conditions, and image acquisition techniques may affect the reproducibility and performance of UVF dermoscopy in real-world clinical settings. Finally, external validation across geographically diverse populations and healthcare systems is necessary to establish the generalizability and broader clinical applicability of the findings.

## Conclusions

Ultraviolet-induced fluorescence dermoscopy demonstrated high sensitivity and high negative predictive value in the evaluation of superficial cutaneous fungal infections. The technique provides rapid, non-invasive bedside assessment and may facilitate earlier recognition of fungal disease as well as more accurate site selection for KOH sampling. Although specificity was moderate due to fluorescence overlap with inflammatory and bacterial dermatoses, the overall diagnostic performance was very good, with an AUC of 0.896. Therefore, UVF dermoscopy should be considered a valuable adjunctive screening tool rather than a replacement for conventional mycological investigations such as KOH microscopy, fungal culture, or PCR. Further prospective multicenter studies incorporating fungal culture and molecular diagnostics are recommended to validate these findings and establish standardized fluorescence criteria.
